# Highly Pathogenic Avian Influenza A(H5N8) Virus in Gray Seals, Baltic Sea

**DOI:** 10.3201/eid2512.181472

**Published:** 2019-12

**Authors:** Dai-Lun Shin, Ursula Siebert, Jan Lakemeyer, Miguel Grilo, Iwona Pawliczka, Nai-Huei Wu, Peter Valentin-Weigand, Ludwig Haas, Georg Herrler

**Affiliations:** University of Veterinary Medicine Hannover, Hannover, Germany (D.-L. Shin, U. Siebert, J. Lakemeyer, M. Grilo, N.-H. Wu, P. Valentin-Weigand, L. Haas, G. Herrler);; University of Gdańsk, Gdańsk, Poland (I. Pawliczka)

**Keywords:** H5N8 influenza virus, highly pathogenic avian influenza, marine mammals, seal, influenza, viruses, Baltic Sea, Poland, respiratory infections, zoonoses

## Abstract

We detected a highly pathogenic avian influenza A(H5N8) virus in lung samples of 2 gray seals (*Halichoerus grypus*) stranded on the Baltic coast of Poland in 2016 and 2017. This virus, clade 2.3.4.4 B, was closely related to avian H5N8 viruses circulating in Europe at the time.

In 1996, emerging highly pathogenic avian influenza (HPAI) viruses caused outbreaks in domestic poultry in China. The ancestral virus (A/goose/Guangdong/1/1996(H5N1); gs/Gd) and the related reassortant viruses have continued to cause outbreaks in birds and have been associated with human infections. Multiple genetic linages of the hemagglutinin (HA) gene are clustered into 10 clades (*1*). In 2014, gs/Gd-lineage H5Nx HPAI viruses belonging to clade 2.3.4.4 were detected in Eurasia, followed by a novel lineage 2.3.4.4 B of H5N8 viruses detected in wild birds in 2016. This reassortant H5N8 virus is widespread among wild birds worldwide, causing mass deaths in waterfowl, its natural reservoir (*2*). No natural transmission of this virus from birds to marine mammals has been reported.

In 2014, an epizootic among harbor seals infected with avian influenza viruses (AIV) of subtype H10N7 was reported at the coast of northern Europe. Infected seals displayed multifocal pyogranulomatous to necrotizing pneumonia, which led to death (*3*–*5*). Various outbreaks of H3N8, H7N7, and H4N6 low pathogenicity avian influenza (LPAI) viruses have occurred in harbor seals along the New England coast of the United States (*6*). Yet, the exact route of viral transmission from bird to seal remains unclear. Avian, but not human, influenza viruses have been reported to attach to cells of the respiratory tract of seals (*7*). The limited studies do not provide a comprehensive picture about the abundance of avian-type α2,3-linked sialic acid receptor molecules on the airway epithelium of seals (*8*).

## The Study

On November 27, 2016, an immature male gray seal estimated to be 20 months old was found dead on the Baltic coast of Poland; it was in a state of initial decomposition and displayed poor nutritional status. Pathologic findings included a parasitic infestation (*Halarachne halichoeri*) in the nasal cavity, lung, and gastrointestinal tract; agonal changes, including pulmonary edema and emphysema, were observed. A second male seal with estimated age of 2 months was found on April 21, 2017; it was emaciated and showed several signs of trauma. It had mild to severe parasitic infestation in the digestive tract. Bacteriologic investigation provided evidence for the presence of several different bacteria. 

We obtained a lung sample from each animal for virologic analysis. PCR results were negative for phocine distemper virus and phocine herpesvirus 1 in the lung tissues of both animals. However, we detected influenza A virus RNA using a real-time reverse transcription PCR targeting the NP gene (provided by Timm Harder, Friedrich-Loeffler-Institut, Greifswald-Riems, Germany). We isolated and propagated the virus from the lung of the older seal by using MDCK cells and designated the isolate as A/gray seal/BalticPL/361-10/2016 (GISAID [https://www.gisaid.org] accession no. EPI_ISL_322984). We sequenced HA, NA, and internal segments using Sanger sequencing. The isolation of the virus from the other animal failed; however, we were able to perform direct sequencing of the HA and NA genes (A/gray seal/BalticPL/361-13/2017; GISAID accession no. EPI_ISL_362127). The results confirmed that both animals were infected by the same H5N8 virus (H5N8/seal) with a multibasic cleavage site of PLREKRRKR/GLF in its HA protein, which fits the consensus sequence of a clade 2.3.4. HPAI virus (*1*). Phylogenetic analysis of the HA and NA segments using the GISAID EpiFLU database further revealed that the isolate belonged to the clade 2.3.4.4 B group of H5 HPAI viruses (Figure). Results of a homology BLAST search (https://blast.ncbi.nlm.nih.gov/Blast.cgi) showed that this H5N8/seal virus had a nucleotide homology of 99.7%–100% to viruses that were circulating in aquatic wild bird species during the avian influenza outbreaks in 2016 and 2017. Alignment of viral RNA using ClustalW (http://www.clustal.org) showed that no coding mutation was found in the H5N8/seal virus compared with A/tufted duck/Germany/AR8444/2016 (H5N8).

**Figure:**
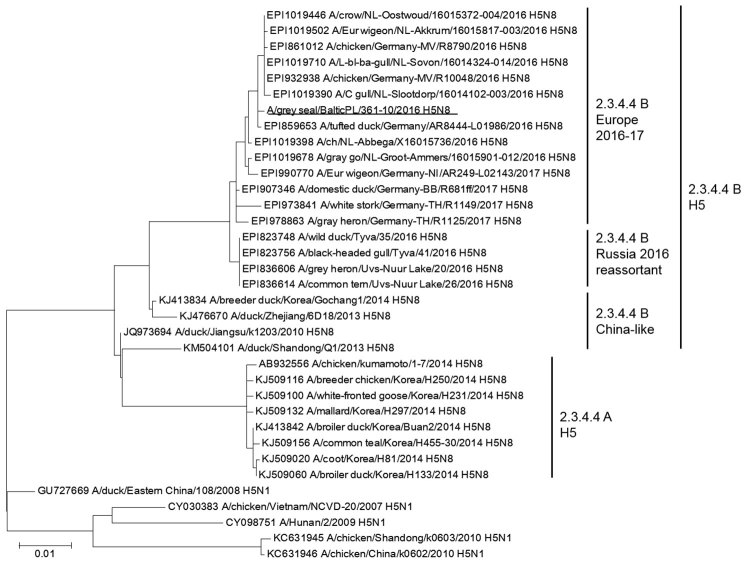
Maximum-likelihood phylogenetic tree for the hemagglutinin genes of a highly pathogenic avian influenza A(H5N8) virus isolated from a seal in the Baltic Sea region of Poland (underlined) and reference sequences. Different clades and the subclades of 2.3.4.4 are marked. Accession numbers for reference sequences are provided; numbers beginning with EPI are from the GISAID EpiFLU database (https://www.gisaid.org), others from GenBank. Scale bar indicates nucleotide substitutions per site.

## Conclusions

We report the case of a clade 2.3.4.4 B group HPAI H5N8 virus able to infect marine mammals. The isolated H5N8/seal virus showed 99%–100% identity to the avian strains that were circulating in Europe during 2016–2017. HPAI H5N8 2.3.4.4 B virus infections are associated with severe symptoms in infected waterfowl or wild birds. The AIV AR8444 strain in the EpiFLU database with the highest homology to H5N8/seal was isolated from a dead tufted duck found in Lake Plön, Schleswig-Holstein, in northern Germany. Experimental infection of ducks with the AR8444 strain resulted in a mortality rate of 33% 4–8 days postinfection (*9*).

Pinnipeds, including seals, are susceptible to various viral pathogens, such as influenza A and B viruses, morbillivirus, and herpesvirus. Most of the influenza viruses isolated from harbor seals were closely related to avian influenza viruses, such as H7N7 (*10*), H3N8 (*8*), and H10N7, of which there was an outbreak in 2014 (*5*). However, the exact transmission pathway of AIV from birds to seals is still unknown, and to our knowledge, HPAI viruses have not been isolated from seals.

We describe findings from 2 dead seals collected during the avian influenza outbreaks of 2016 and 2017 by the Prof. Krzysztof Skóra Hel Marine Station; these 2 were positive for AIV by real-time reverse transcription PCR. Examination of the lungs by gross pathology and histopathology did not reveal any suspicious lesions that indicated an influenza virus infection. No evidence of a related outbreak or mass deaths has been observed in the Baltic seal population. The positive samples appear to be the result of HPAI spillovers from birds to the gray seals. The finding of 2 seals infected 5 months apart suggests that such cross-species transmissions can occur sporadically, but we cannot exclude the possibility of seal-to-seal transmission. There is no evidence that this virus is highly pathogenic for seals.

Studies have shown that some mutations known to enhance the transmissibility of H5N1 HPAI viruses may increase the ability of LPAI viruses to be transmitted from bird to marine mammal (*11**–**13*). These factors include the change of sialic acid receptor binding affinity (*11*) and adaptive mutations in the vRNP complex for replication and virus spread in the seal population (*12*). In the H5N8/seal isolate, we detected no molecular markers previously associated with the transmission of avian-derived influenza viruses to marine mammals (*13*) in the viral PB2, PB1, PA, or HA segments (Table). Thus, it appears that no adaptive mutations have occurred in the gray seal analyzed in this study.

**Table:** Molecular markers for enhancing interspecies transmission ability of highly pathogenic avian influenza A(H5N8) virus to seals, Poland*

Subtype	Location	Year isolated	Amino acid position									
			PB2				PB1		PA		HA†	
			17C	E627K	D701N		453S		192H		226L	228S
H5N8‡	Baltic Sea	2016	R	E	D		A		R		Q	G
H10N7§	North Sea	2015	C	E	D		S		H		L	G
H3N8¶	North Atlantic Ocean	2011	R	E	N		A		R		Q	G
H4N5#	North Atlantic Ocean	1982	R	E	D		A		R		Q	G
H7N7**	North Atlantic Ocean	1980	R	E	D		A		R		Q	G

*HA, hemagglutinin; PB1, polymerase basic 1; PB2, polymerase basic 2; PA, polymerase.  †All HA genes are in H3 numbering.  ‡Strain destination: A/gray seal/BalticPL/361–10/2016 (this study). §Strain destination: A/harbor seal/Netherlands/PV14–221_ThS/2015. ¶Strain destination: A/harbor seal/New Hampshire/179629/2011. #Strain destination: A/harbor seal/Massachusetts/133/1982. **Strain destination: A/harbor seal/Massachusetts/1/1980.

Most reports on influenza viruses in seals are related to outbreaks in harbor seals and not gray seals. However, seroprevalences against H10N7 influenza A virus were described in gray seals in the Netherlands (*14*). In addition, influenza A virus matrix RNA (without further characterization) was detected in swab samples of 9.0% of apparently healthy weaned gray seal pups live-captured in the North Atlantic (*15*). In adult seals, seroprevalence was 50%; the authors suggest a possible role of gray seals as a wild reservoir of influenza A virus. These reports indicate that the gray seal can be infected by influenza viruses. Because we describe a naturally occurring spillover of HPAI virus to a marine mammal, future surveillance programs should continue to monitor gray seals and harbor seals as possible reservoirs of AIV.

Appendix Additional information used in the study of highly pathogenic avian influenza A(H5N8) virus in seals, Baltic Sea.
